# Targeting Cell Cycle in Breast Cancer: CDK4/6 Inhibitors

**DOI:** 10.3390/ijms21186479

**Published:** 2020-09-04

**Authors:** Michela Piezzo, Stefania Cocco, Roberta Caputo, Daniela Cianniello, Germira Di Gioia, Vincenzo Di Lauro, Giuseppina Fusco, Claudia Martinelli, Francesco Nuzzo, Matilde Pensabene, Michelino De Laurentiis

**Affiliations:** 1 Department of Breast and Thoracic Oncology, Division of Breast Medical Oncology, Istituto Nazionale Tumori IRCCS “Fondazione G. Pascale”, 80131 Naples, Italy; m.piezzo@breastunit.org (M.P.); s.cocco@breastunit.org (S.C.); r.caputo@breastunit.org (R.C.); d.cianniello@breastunit.org (D.C.); germiradigioia@gmail.com (G.D.G.); dilaurovincenzo87@gmail.com (V.D.L.); g.fusco@istitutotumori.na.it (G.F.); f.nuzzo@istitutotumori.na.it (F.N.); m.pensabene@istitutotumori.na.it (M.P.); 2 Department of Clinical Medicine and Surgery, University of Naples “Federico II”, 80131 Naples, Italy; claudia.martinelli1993@gmail.com

**Keywords:** cell cycle, cyclin-dependent kinase, cancer, metastatic breast cancer, hormone therapy, CDK4/6 inhibitors, hormone receptors, therapies

## Abstract

Deregulation of cell cycle, via cyclin D/CDK/pRb pathway, is frequently observed in breast cancer lending support to the development of drugs targeting the cell cycle control machinery, like the inhibitors of the cycline-dependent kinases (CDK) 4 and 6. Up to now, three CDK4/6 inhibitors have been approved by FDA for the treatment of hormone receptor-positive (HR+), HER2-negative metastatic breast cancer. These agents have been effective in improving the clinical outcomes, but the development of intrinsic or acquired resistance can limit the efficacy of these treatments. Clinical and translational research is now focused on investigation of the mechanism of sensitivity/resistance to CDK4/6 inhibition and novel therapeutic strategies aimed to improve clinical outcomes. This review summarizes the available knowledge regarding CDK4/6 inhibitor, the discovery of new biomarkers of response, and the biological rationale for new combination strategies of treatment.

## 1. Introduction

Hormone receptor (HR) positive breast cancer (BC) subtypes (luminal A and B) represent approximately 60%–75% of all breast cancers and respond well to endocrine therapy (ET), in both adjuvant and metastatic setting. However, while some patients show de-novo resistance (primary) virtually all the remaining ones develop acquired resistance (secondary) to ET, which ultimately leads, after 1–2 further attempts with other endocrine-based regimens (± targeted agents, such as everolimus), to chemotherapy-based regimens [1]. Several aberrations and signaling pathways are involved in endocrine resistance, such as PI3K/Akt/mTOR pathway, cyclin D/CDK/pRb pathway, ESR1 gene mutations, cross talk between ER and growth factors receptors signaling, and epigenetic alterations [2,3]. It is well-known that dysregulation of cyclin D-CDK4/6-pRb pathway represents a key mediator of endocrine resistance in HR-positive BC [4,5]. Targeting CDK4/6 with specific inhibitors in HR-positive BC has proven effective both in the preclinical and the clinical setting. Recently, US Food and Drug Administration (FDA) and European Medicine Agency (EMA) approved three orally highly selective inhibitors of CDK4/6 for HR-positive advanced or metastatic BC, namely palbociclib (PD0332991), ribociclib (LEE011), and abemaciclib (LY2835219). The aim of this review is to summarize the pharmacological background of CDK4/6 inhibitors (CDK4/6i), the latest evidence of their efficacy for the treatment of BC, their potential use in combination with other targeted therapies and the role of predictive biomarkers, focusing on affinities and differences among these three agents.

## 2. The Role of Cyclin-Dependent Kinases 4/6 (CDK4/6) and Cell Cycle Control in Breast Cancer

**Figure 1:**
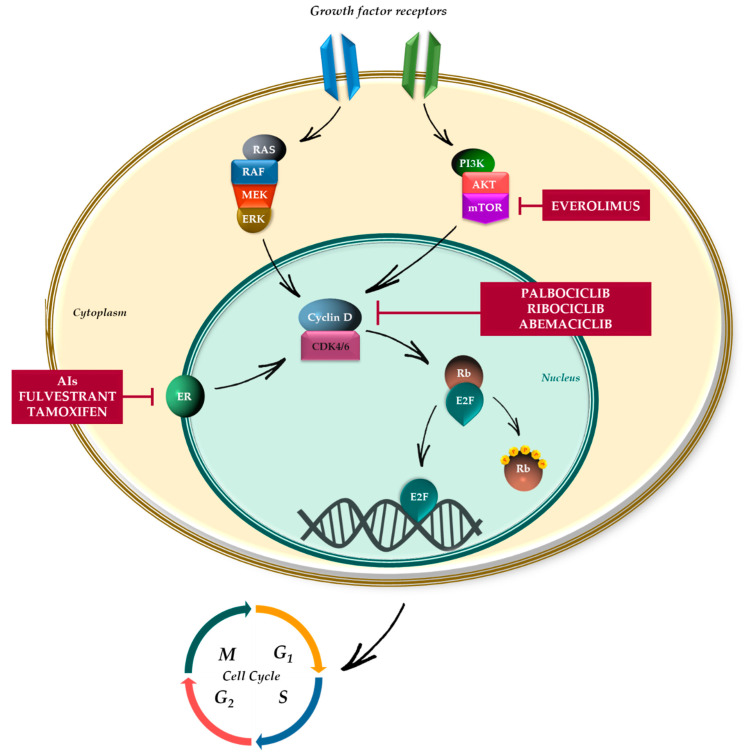
Mechanism of action of CDK4/6 inhibitors. Activation of upstream signaling pathways, such as MAPK, PI3K, and ER, regulate the progression of cell cycle by promoting the formation of complex cyclin D-CDK4/6, which selectively phosphorylates and inactivates pRb protein. Rb proteins limit the expression of many E2F target genes which are involved in cell cycle progression, DNA replication, and mitotic progression. CDK4/6 inhibitors (palbociclib, ribociclib, and abemaciclib) selectively inhibit the downstream CDK4/6-mediated phosphorylation of Rb, leading to cell cycle arrest in G0/G1 phase. Combination strategies are focused on dual blockade of CDK4/6 and upstream signaling, mainly mediated by ER, MAPK pathway and PI3K/AKT/mTOR pathway.

The maintenance of tissue homeostasis relies on two major physiologic processes: cell division and death. Through these two mechanisms, cells are able to respond to tissue damage and proliferate according to tissue requirements, avoiding over-proliferation and limiting the potential for cancer. The majority of differentiated cells in adult tissues are maintained in a dormancy status, the G0 state, waiting for entering the cell cycle. The G0 state can be either transient (quiescence) or permanent (senescence); quiescent cells can re-enter the cell cycle when receiving mitogenic stimuli, such as growth factors or hormonal stimuli. The cell cycle process consists in four ordered phases, highly conserved and controlled, named G0/G1 (gap 1), S (DNA synthesis), G2 (gap 2), and M (mitosis). The Gap phases (G1 and G2) represent the key regulatory checkpoints, regulated by many cyclins and CDKs, determining whether cells enter into S phase (G1 restriction point) and move forward with mitosis. The mid-G1 phase is governed by CDK4 and CDK6, two serine/threonine kinases the catalytic activity of which is modulated by D-type cyclins (D1, D2, and D3) [1,6]. During the early G1 phase, in response to mitogenic stimuli, cyclins D1, D2, and D3 bind and activate CDK4 and CDK6, subsequently the complex cyclin D-CDK4/6 selectively phosphorylates and inactivates members of Retinoblastoma-associated proteins (pRb), such as p110 (encoded by *RB1*), the related pocket protein p107 (encoded by *RBL1*) and p130 (encoded by *RB2*) [7,8]. Rb proteins are transcriptional co-repressors and limit the expression of many E2F target genes which are involved in cell cycle progression, DNA replication, and mitotic progression [9,10]. The hyperphosphorylation of Rb limit this transcriptional repression by reducing the affinity for E2F and leading to release of E2F transcription factors, allowing for transcription of CDK2, E-type cyclins, and other proteins which in turn constitute a complex, able to further phosphorylates Rb, promoting S phase entry [11,12,13]. The kinase activity of CDK4/6 is negatively regulated by two families of CDK inhibitors (CDKi), able to bind the ATP-binding pocket of CDK and to inhibit the downstream CDK4/6-mediated phosphorylation of Rb; this endogenous inhibition of CDK4/6 potently arrest cell cycle but requires functional Rb protein (Figure 1) [14,15]. The two families of CDKi, having different structure and CDK specificity, are the CDK-interacting protein/kinase inhibitory protein (CIP/KIP) family, including p21^CIP1^ (*CDKN1A*), p27^KIP1^ (*CDKN1B*), and p57^KIP2^ (*CDKN2D*) proteins, and the inhibitor of CDK4 (INK4) family, including p16^INK4A^ (*CDKN2A*), p14^ARF^ (CDKN2A), p15^INK4B^ (*CDKN2B*), p18^INK4C^ (*CDKN2C*), and p19^INK4D^ (*CDKN2D*) proteins. The INK4 family proteins specifically interact with the catalytic domains of CDK4/6 inhibiting their association with D-type cyclins and suppressing the kinase activity, while the CIP/KIP family proteins have both inhibitory and sometimes activating effects, interfering with the activity of all cyclin-CDK complexes [16,17,18].

Transcription of D-type cyclins is closely linked to multiple pathways, as well as CDK4/6 activity that acts as a sensor linking multiple signaling pathways to the initiation and progression of the cell cycle [19,20,21]. Dysregulation in cyclin/CDK/Rb pathway is frequent in many type of human cancers, including breast cancer (BC), in which CDK4/6 have been identified as key drivers of proliferation in HR-positive BC [18]. Amplification and overexpression of *CCND1* oncogene, encoding cyclin-D1 protein, is frequent in BC, more specifically within luminal A (29%), luminal B (58%), and human epidermal growth factor receptor 2 (HER2) enriched (38%) subtypes, while *CCND2* or *CCND3* amplification is rare. Similarly, *CDK4* gene amplification has been observed more specifically in 14% of luminal A, in 25% of luminal B, and in 24% of HER2 enriched tumors. The activity of major tumor suppressors, *RB1* and *TP53* is generally conserved in luminal and HER2 enriched subtypes, while about 20% of triple negative BC (TNBC) lack functional Rb protein, making the use of CDK4/6 inhibitors more challenging; however, the luminal androgen receptor (LAR) subgroup, a subtype of TNBC, might be sensitive to inhibition of CDK4/6 pathway due to the association of AR expression and *RB1* expression [4,22].

Luminal tumors are generally characterized by an enhanced expression of cyclin D, via estrogen receptor activation that bind directly to the *CCND1* promoter, enhancing the cyclin D1 expression and modulating the mitosis process [23,24]. In addition, also the RAS-RAF-MEK-ERK pathway and the HER2-PI3K-AKT axis play a significant role in regulating cyclin D1 expression [25,26].

Because of the coordination function of cyclin D and CDK4/6 in cell cycle regulation, this axis represented an attractive target for the development of therapeutic strategies. In this scenario, the development of CDK4/6 inhibitors has been the most interesting finding, since the vulnerability of cancer cells has been fought maintaining a tolerable toxicity profile.

## 3. Pharmacological Activity of CDK4/6 Inhibitors

Multiple generations of CDK inhibitors drugs have been developed during the past years. The first generation of CDK-directed drugs (flavopiridol, roscovitine, olomucine) were non-selective pan-inhibitors, which resulted ineffective in clinical trials. Second-generation CDK-directed drugs (dinaciclib, AT7519, R547, SNS-032, BMS-387032, AZD5438, AG-024322), reported high toxicity in phase I/II trials united to not clear mechanism of action, low specificity, and inappropriate CDK family selectivity [27].

A third generation of CDK-directed drugs were developed to be selective versus CDK4/6 kinases. In particular, three CDK4/6 inhibitors, palbociclib, ribociclib, and abemaciclib have been tested in clinical trials with demonstrated efficacy and tolerable toxicity profile in breast cancer patients [28]. Palbociclib, Ribociclib, and Abemaciclib are orally bioavailable, highly selective small molecule inhibitor of CDK4/6. Palbociclib has selective activity for CDK4/cyclin D1 kinase with an IC50 of 0.011 μmol/L and little or no activity against a large panel of 274 other protein kinases, including other CDKs and a wide variety of tyrosine and serine/threonine kinases [29]. Ribociclib inhibits the CDK4/cyclin D1 and CDK6/cyclin D3 enzyme complexes with IC50 values of 0.01 and 0.039 μM in biochemical assays, respectively, while showing a high degree of selectivity for CDK4/6 versus other cyclin-dependent kinases. The IC50 values observed for the abemaciclib mesylate (LSN2813542) are 1.6 nM, 2.0 nM, 180 nM, for the inhibition of CDK4, CDK6, and pRb phosphorylation, respectively [30]. Palbociclib and ribociclib have a similar structure optimized for high selectivity toward CDK4/6, while abemaciclib has a different chemical structure that allows inhibition of other kinases, particularly CDK9, although this does not translate in CDK9 inhibition in cellular models [28,31]. Chen et al. reported that the three inhibitors trigger the inhibition of proliferation in pRb-competent cells with different potency. Palbociclib and abemaciclib resulted more effective in the inhibition of phosphorylation of serin 807 and serin 780 of RB compared to Ribociclib, evaluated in different breast cancer cell lines. The combination with ER antagonists resulted particularly effective with palbociclib and ribociclib while abemaciclib had significant single agent activity [32].

Further in vitro lines of evidence have shown that the anti-tumor activity of palbociclib is selective for Rb-positive tumors since Rb negative cell lines, such as MDA-MB-468 breast carcinoma and the DU-145 prostate tumor models, do not responding to this compound [33,34]. Palbociclib also exerted a potent anti-tumor activity in vivo, inducing a robust growth suppression in xenografts derived from different tumors types, including breast cancer [28,29]. Ribociclib has demonstrated in vivo anti-tumor activity in different tumor xenograft models including breast, melanoma, neuroblastoma, malignant rhabdoid, lung, pancreas, and hematological malignancies [35,36,37,38]. In addition, ribociclib has shown anti-tumor activity when combined with targeted agents which inhibit signaling pathways known to regulate D-cyclin levels, including inhibitors of the RAF, mitogen-activated protein kinase kinase (MEK), phosphoinositide 3-kinase (PIK3), and mammalian target of rapamycin (mTOR) pathways [35,39,40]. In ER-positive (ER+) breast cancer xenograft models, combinations of ribociclib with endocrine therapy such as letrozole, fulvestrant, and tamoxifen demonstrate a statistically significant, strong, and sustained antitumor activity compared to endocrine therapy or ribociclib alone [41]. Abemaciclib mesylate showed significant inhibition of tumor growth in different murine xenograft models of human cancer, such as Colo-205 (colorectal cancer), NCI-H460 (NSCLC), U87 MG (glioblastoma), and JeKo-1 (MCL), all characterized by functional Rb tumor suppressor protein. The growth inhibition resulted dose-dependent (from 15 to 100 mg/kg following daily oral administration for 21 days) and was associated with a sustained inhibition of pRb, TopoIIα, and pHH3 [31,42,43].

## 4. Efficacy and Safety Profile of CDK4/6 Inhibitors in HR-Positive, HER2 Negative Metastatic BC

The inclusion of CDK4/6 inhibitors in combination with endocrine therapy in international treatment guidelines, both as initial therapy and after disease progression following ET, represents the most relevant advance in the management of HR-positive, HER2-negative advanced, or metastatic BC over the past decade [44,45,46]. Currently three selective CDK4/6 inhibitors (palbociclib (PD0332991), ribociclib (LEE011), abemaciclib (LY2835219)) have been granted approval by both FDA and EMA for the treatment of HR-positive, HER2-negative metastatic BC.

Palbociclib (Ibrance, Pfizer, NY, NY, USA) is the first-in-class CDK4/6i receiving an accelerated approval from FDA in February 2015, in combination with letrozole as initial therapy for postmenopausal women HR-positive, HER2-negative advanced, or metastatic BC, based on the results from the phase II trial PALOMA-1/TRIO-18, which showed a significant benefit in terms of progression-free survival (PFS) for combination therapy with palbociclib and letrozole over letrozole alone (median PFS 20.2 vs. 10.2 months, hazard ratio (HR) 0.49, *p* = 0.0004) [47]. Nevertheless, the trial cannot show a similar statistically significant increase of overall survival (OS), showing a median OS of 37.5 months in the palbociclib-letrozole group, as compared with 34.5 months in the placebo-letrozole group (HR 0.897, *p* = 0.281) [48]. These results were validated by conducting the phase III PALOMA-2 trial, which showed a final PFS of 27.6 months in palbociclib plus letrozolo arm, compared with 14.5 months in letrozole arm (HR 0.563, *p* < 0.001) and a clinical benefit rate (CBR) improved from 70.3% to 84.9% (*p* < 0.001) favoring palbociclib [49,50]. Successively, data from PALOMA-3 trial led to expanded indication of palbociclib in combination with fulvestrant for the treatment of both postmenopausal and pre-perimenopausal women in which disease progressed following ET. The addition of palbociclib to fulvestrant significantly prolonged PFS from 4.6 to 9.5 months (HR 0.46, *p* < 0.001), without any statistically significant prolongation of OS (HR 0.81, *p* = 0.09) [51,52,53,54]. In April 2019, the indication of palbociclib was further expanded to male patients, based on evidence from real world studies [55]. Finally, based on results from FALCON trial demonstrating the superiority of fulvestrant to anastrozole in the same population of PALOMA-1 and PALOMA-2 trials, the phase II PARSIFAL study (NCT02491983) investigated the choice of best endocrine agent to combine with palbociclib in this first-line setting; results were presented at last Annual Meetings of the American Society of Clinical Oncology (ASCO) and showed no statistically significant differences between the two groups (median PFS was 27.9 months in palbocilib plus fulvestrant arm versus 32.8 months in palbocilcib plus letrozole arm, HR 1.1, *p* = 0.321), suggesting that fulvestrant can be a reasonable alternative to letrozole in combination with palbociclib as first line treatment for endocrine sensitive metastatic BC (relapse > 12 months after the end of adjuvant ET or diagnosed with “de novo” metastatic disease) [56].

Ribociclib (Kisqali, Novartis, Basel, Switzerland) is the second CDK4/6i receiving the FDA approval in March 2017 as the first line therapy for postmenopausal women with HR-positive, HER2-negative advanced, or metastatic BC in combination with an aromatase inhibitor (AI). Its first approval is based on results from phase III MONALESA-2 trial, showing a significant prolongation of PFS of 25.3 months in the combination arm (ribociclib plus letrozole) and 16.0 months in letrozole alone arm (HR 0.568, *p* < 0.001) [57,58]. In the phase III MONALEESA-3 trial, ribociclib was also investigated in combination with fulvestrant as the first or second line treatment in postmenopausal HR-positive, HER2-negative metastatic BC patients. In contrast to PALOMA-3 study, MONALEESA-3 included ET treatment naïve patients (about 50% of enrolled patients) or late relapsed patients (>12 months from completion of adjuvant ET), showing a PFS improvement of 20.5 vs. 12.8 months, favoring ribociclib (HR 0.593, *p* < 0.001) and a median OS not reached in the ribociclib-fulvestrant group and 40 months in the placebo-fulvestrant group (HR 0.724, *p* = 0.004) [53,59,60,61]. In addition, ribociclib was also investigated in combination with ET plus goserelin for the treatment of peri/premenopausal women who received up to one line of chemotherapy and no previous ET for advanced/metastatic disease (phase III trial MONALEESA-7), showing a significant prolonged PFS (23.8 vs. 13.0 months, HR 0.55, *p* < 0.0001) and OS (not reached in ribociclib group vs. 40.9 months in the placebo group, HR for death 0.71, *p* = 0.009) [62,63].

Abemaciclib (Verzenio, Lilly, Indianapolis, IN, USA) is the third CDK4/6i granted quick FDA approval in September 2017, based on the results from phase III MONARCH-2 trial, aimed to investigate abemaciclib in combination with fulvestrant in women with HR-positive/HER2-negative advanced BC in which disease had progressed after ET. PFS was significantly improved in abemabiclib plus fulvestrant arm compared to fulvestrant alone arm (median PFS 16.4 vs. 9.3 months, HR 0.553, *p* < 0.001), just as OS that achieved 46.7 months in combination arm versus 37.3 months in fulvestrant arm (HR 0.757, *p* = 0.01) [64,65]. In addition, the efficacy of abemaciclib plus non-steroidal AI (NSAI) in women who had no prior systemic treatment for advanced/metastatic BC was confirmed by phase III MONARCH-3 trial (median PFS 28.2 vs. 14.8 months, HR 0.54, *p* < 0.001) [66,67]. Abemaciclib has also been approved as a monotherapy for patients with HR-positive/HER2-negative metastatic BC who have previously received endocrine therapy and chemotherapy, based on results from single-arm phase II MONARCH-1 trial [68].

In summary, palbociclib and ribociclib showed similar efficacy profile and similar prescribing indications: in combination with AI as the first line treatment and in combination with fulvestrant as the subsequent line of therapy in both pre and post-menopausal patients with HR-positive, HER2-negative advanced/metastatic BC. In contrast, the prescribing indication for abemaciclib is quite different, it can be used in combination with fulvestrant for the treatment of women with HR-positive, HER2-negative advanced/metastatic BC previously progressed on ET, and as monotherapy for patients previously treated with ET and chemotherapy in metastatic setting (Table 1) [55,69,70,71,72,73]. 

Treatment with CDK4/6i in combination with ET is generally safe and well tolerated. Toxicities are easily treatable and can be managed with dose adjustment and supportive care. Hematological toxicity is commonly seen with all three inhibitors, but some hematological adverse events (AEs) are more frequent with palbociclib and ribociclib rather than abemaciclib, for example grade 3–4 neutropenia occurs in 66% and 60% of patients treated with palbociclib and ribociclib respectively, but only in 22% of patients treated with abemaciclib. It is well-known that all CDK4/6 inhibitors play a key role in proliferation of hematological precursors, showing a cytostatic action on neutrophil precursors that results in cellular quiescence; this effect is rapidly recovered when drug is held, that is the reason of intermittent schedule of palbociclib and ribociclib. Palbociclib is administered orally with food, in order to increase the drug exposure, at the dose of 125 mg/day on a 3/1 schedule (21-day on, 7-day off) and if needed dose can be reduced to 100 mg/day and successively at the final dose of 75 mg/day, Ref. [69]. Ribociclib taken orally at the dose of 600 mg/day on a 3/1 schedule (21-day on, 7-day off); dose reduction is allowed at 400 mg/day and 200 mg/day as final dose [70]. Abemaciclib is associated to a lower prevalence of hematological toxicity, being more selective for CDK4 and it can be dosed continuously at the dose of 200 mg twice daily as monotherapy or at the starting dose of 150 mg twice daily in combination with ET [71]. There are some distinct toxicities that are peculiar to the different CDK4/6i: ribociclib has been associated with high prevalence of hepatotoxicity and reversible prolongation of QT interval, while abemaciclib results in higher prevalence of diarrhea, fatigue, venous thromboembolic events, and increased levels of serum creatinine [57,66,68,74].

Two additional CDK 4/6 inhibitors are currently undergoing clinical development: lerociclib (G1T38) and Trilaciclib (G1T28). Lerociclib is a differentiated oral CDK4/6i, currently being evaluated in a phase I/II trial (NCT02983071) in combination with fulvestrant in HR-positive, HER2-negative metastatic BC patients; preliminary results presented at San Antonio Breast Cancer Symposium (SABCS) in December 2019 showed safety and efficacy profile consistent with other CDK4/6 inhibitors [75].

Trilaciclib is a first-in-class “Breakthrough Therapy,” formulated for intravenous delivery, designed to preventing toxicities induced by chemotherapy. It inhibits the CDK4/6 pathway, targeting haemopoietic stem and progenitor cells and lymphocytes; it is able to transiently maintain immune cells and hemopoietic stem and progenitor cells in G1 arrest, protecting the immune cells and bone marrow from chemotherapy-induced damage. Trilaciclib has been tested in small cell lung cancer (positive results) and in a randomized trial in metastatic TNBC [76]. Additional trials in BC are planned in 2020.

## 5. Biological Rationale for Novel Combination Strategies

In HER2-positive cells, the HER2/Akt pathway is a negative regulator of the CDK inhibitor p57 (Kip2), which leads to increased cell proliferation [77]. In this context, preclinical data suggest that CDK4/6 inhibition may be effective in HER2-positive breast cancer and could restore sensitivity to anti-HER2 therapies [78]. In vitro studies suggested that cyclin D1/CDK4 complex could be responsible of resistance to anti-HER2 therapy and CDK4/6 inhibitors were effective as single agents or in combination with trastuzumab to restore sensibility to anti-HER2 therapy [79]. At the same time, preclinical models of acquired resistance to HER2-targeted therapies, showed an increased activation of Cyclin D1 and CDK4/6 inhibition was effective at blocking proliferation [80]. In addition, Zhang et al. reported that palbociclib and pyrotynib, a pan-HER2 inhibitor, were highly synergistic in inhibiting cancer proliferation in in vitro and in vivo HER2 models [81]. Because of these preclinical studies, clinical studies combining CDK4/6 inhibitors and anti-HER2 therapy have rapidly emerged [82].

Activation of the phosphoinositide 3-kinase (PI3K) pathway occurs frequently in breast cancer, potentially leading to resistance to endocrine therapy. However, clinical results of single-agent PI3K inhibitors have been modest to date [83]. Preclinical studies showed that PI3K/Akt/mTOR inhibitors sensitized ER-positive cell lines to CDK4/6 inhibition, and the triple combination with ET, was more effective than double combinations [84]. At the same time, the combination of ribociclib plus the α-specific PI3K inhibitor, alpelisib (BYL719), demonstrated synergistic activity in PIK3CA mutant BC cell lines. Importantly, the combination of PI3K and CDK 4/6 inhibitors overcome intrinsic and adaptive resistance to PI3K inhibitors leading to tumor regression in PIK3CA mutant xenografts [85]. Finally, it has been shown that a combination of palbociclib and dual mTOR kinase inhibitor, MLN0128, synergistically inhibited the proliferation and induced G1 cell cycle arrest in pRb-expressing HR-negative breast cancer cell lines [86].

Other findings reported that the LAR subgroup of triple negative breast cancer (TNBC) could over-express Rb protein, thus becoming sensitive to CDK4/6 inhibitors [87,88,89,90]. Interestingly, preclinical studies showed that CDK4/6 inhibitors could also regulate mitogenic kinase signaling, inducing senescence and promoting anti-tumor immunity [91,92], providing the rationale of combinations with immunotherapy agents. In particular, by suppressing the Rb-E2F axis, CDK4/6 inhibitors could enhance antigen presentation [93]. CDK4/6 inhibitors could repress immunosuppressive regulatory T cells (Tregs) and enhance effector T cells response by upregulating the level of cytokines (IL-2) in the tumor microenvironment [94]. Cyclin D-CDK4 complex was also reported to play a role in reducing the expression of PD-L1; therefore CDK4/6 inhibitors could promote expression of PD-L1, causing tumor immune evasion [95,96]. These findings suggested that CDK4/6 inhibitors showed combinatorial benefit when combined with anti-PD-L1 therapy. Ongoing clinical studies will shed light onto the mechanisms of response CDK 4/6 and immunotherapy combination.

The effects of combining CDK 4/6 inhibitors with chemotherapy has also been explored in preclinical studies, with controversial results. The combination of CDK4/6 inhibitors with paclitaxel promoted cell death in HR-positive and TNBC models [97], while combination of palbociclib and carboplatin resulted in decreased antitumor activity compared with carboplatin alone, only in Rb-competent mice [98]. In TNBC cell lines, palbociclib was tested in combination with doxorubicin or paclitaxel, showing that these combinations resulted in cell cycle arrest without cell death in Rb-dependent cells, suggesting that Palbociclib-induced cytostatic effect could interfere with the cytotoxic effect of the chemotherapeutic agent [99]. However, the combination of CDK 4/6 inhibitors with chemotherapy could perturb the cell cycle regulation, therefore the possibility of sequential treatment with CDK 4/6 inhibitors and cytotoxic therapy should be considered [100]. The simultaneous combination of palbociclib and paclitaxel seems to exert an antagonistic effect in Rb-positive TNBC cell models, while the sequential treatment can inhibit cell proliferation and increase cell death more efficiently than single treatment [101]. In this context, trilaciclib has been reported not to decrease chemotherapy efficacy in CDK4/6-dependent xenograft and PDX models [102].

## 6. Novel Treatment Strategies

### 6.1. Neoadjuvant/Adjuvant Therapy

Since the first approval of palbociclib, researchers are focusing on treatment of early BC, trying to demonstrate a benefit with the use of the CDK4/6 inhibitor in addition to ET in this setting. Palbociclib showed a potent antiproliferative effect when administered as a neoadjuvant therapy for stage II/III HR-positive BC in addition to anastrozole, as suggested from the phase II, single arm NeoPalAna study (NCT01723774): patients were stratified by PIK3CA mutational status and received four weeks of anastrozole, followed by four cycles of anastrozole plus palbociclib; the rate of complete cell cycle arrest (CCCA: central Ki67 ≤ 2.7%) after 15 days of combination therapy was 87% versus 26% at day 1 (*p* < 0.001), suggesting that 84% of patients who were resistant to anastrozole alone could become sensitive with the addition of ribociclib. However, after palbociclib washout (suppressed by cycle 5), ki67 recovered at surgery, suggesting that prolonged administration may be necessary to maintain its effect [103]. The phase II PALLET study also demonstrated that patients treated with palbociclib plus letrozole in neoadjuvant setting achieved CCCA in 90% versus 59% of patients treated with letrozole monotherapy (*p* < 0.001), but no significant improvement in clinical response rate was observed (54.3% vs. 49.5%, *p* = 0.20) [104]. Recently, the phase III PALLAS trial (NCT02513394), investigating the adjuvant treatment with palbociclib plus ET for stage II/III HR-positive BC, failed to meet its primary endpoint of invasive disease-free survival (iDFS) during the preplanned interim analysis. Full results are going to be released in the next months. Furthermore, the results from phase III PENELOPE-B study (NCT01864746), evaluating the addition of palbociclib to standard ET as post-neoadjuvant treatment of HR-positive BC patients with a high risk of relapse, are expected later this year.

Ribociclib has been investigated in neoadjuvant setting in the phase II FELINE trial (NCT02712723), which evaluated letrozole plus ribociclib compared with letrozole plus placebo in postmenopausal patients with HR-positive, HER2-negative stage II/III BC. Patients were randomized 1:1:1 to either placebo plus letrozole, intermittent ribociclib plus letrozole, or continuous ribociclib plus letrozole. The primary endpoint was preoperative endocrine prognostic index (PEPI) score of 0 at surgery; the PEPI score should identify patients who are at a low risk for recurrence after neoadjuvant ET and results from FELINE trial that did not meet the primary endpoint, showing that the early suppression of Ki-67 achieved at day 14 in the combination arms was not confirmed at the time of surgery. Additionally, continuous and intermittent dose of ribociclib seem to have similar efficacy but different toxicity profiles. [105]. The adjuvant phase III trial NATALEE, aimed to delay acquired resistance to ET and to improve invasive disease-free survival (iDFS), compares the efficacy and the safety of ribociclib plus standard ET versus ET alone as adjuvant therapy for HR-positive, HER2-negative early BC and is still ongoing [106].

Abemaciclib was investigated as neoadjuvant therapy in combination with anastrozole in the phase II neoMONARCH trial, showing that abemaciclib, alone or in combination with anastrozole, led to potent cell-cycle arrest after 2 weeks of treatment compared with anastrozole alone, 58%–68% of patients treated in the abemaciclib arms versus 14% of patients treated with anastrozole alone achieved CCCA (*p* < 0.001) and a pCR rate of 4% with abemaciclib plus anastrozole. The study also assessed gene expression changes related to cell proliferation and immune response, showing that the combination therapy inhibited cell-cycle processes and estrogen signaling and resulted in increased cytokine signaling and adaptive immune response indicative of enhanced antigen presentation and activated T-cell phenotypes [107]. Recently, Eli Lilly Company announced that phase III MONARCH-E trial (NCT03155997) met its primary endpoint of invasive disease-free survival (iDFS), showing that the combination of abemaciclib and standard ET led to a significant decrease in the risk of BC recurrence or death compared with adjuvant ET alone in patients with high-risk node-positive HR-positive, HER2-negative early BC. A total of 5637 patients were randomized to receive abemaciclib (150 mg twice daily) in combination with standard ET, treatment with abemaciclib was continued for 2 years while ET was administered for a minimum of 5 years. The study is also investigating distant relapse-free survival, overall survival, safety, pharmacokinetics, and health outcomes. Detailed results will be presented later in 2020.

In summary, based on available results, abemaciclib seems to be the only CDK4/6 inhibitor to achieve a statistically significant reduction in the risk of recurrence in this patient population but data for ribociclib are awaited in the next few years. However, these represent early results and should be interpreted cautiously; differences in trial design and in characteristics of population enrolled and availability of full results will allow a better understanding of the clinical impact of CDK4/6 inhibitors in the early BC setting.

### 6.2. Comparison with Chemotherapy

Chemotherapy is still the standard treatment in the neoadjuvant setting and a key question is whether a combination of a CDK4/6i and ET can replace it, thus reducing the burden of toxicity and possibly improving clinical outcomes.

The randomized, phase II NeoPAL study (NCT02400567), examined neoadjuvant letrozole plus palbociclib versus chemotherapy in patients with stage II/III, HR-positive, HER2-negative BC and did not meet the primary endpoint of RCB 0–1 in ≥20% of patients, even if the PEPI score of 0 was reached in 17.6% in the combination arm versus 8% in chemotherapy arm [108].

The phase II CORALEEN study (NCT03248427) compared six 28-days cycles of ribociclib plus letrozole versus chemotherapy (four cycles of doxorubicin plus cyclophosphamide every 21 days followed by weekly paclitaxel for 12 weeks) in women with luminal B stage I-IIIA early BC and then evaluated the proportion of patients with PAM50 low risk-of-relapse (ROR) disease at surgery: results did not show any significant difference between the two groups (low ROR 46.9% vs. 46.1%, pCR rate 2.0% vs. 5.8%, ORR 57.2% vs. 78.8%), moreover, the ribociclib arm resulted in a lower residual cancer burden (RCB) 0–1 rate, of 6.1%, versus 11.8% with chemotherapy [109].

The combination strategy with CDK4/6i plus ET has also been compared with chemotherapy in the metastatic setting. The PEARL study investigated the efficacy of palbociclib plus ET versus capecitabine in postmenopausal women with HR-positive, HER2-negative metastatic BC, previously treated with AI therapy; results did not show any significant differences in terms of progression-free survival (PFS) between the two arms, either in the intention-to-treat (ITT) population or in relevant patients subgroups, such as: (a) women with visceral disease; (b) patients with ESR1 wildtype tumors; patients with DNA expression-based luminal intrinsic subtype breast cancer. However, the Palbociclib plus ET combination was better tolerated [110].

### 6.3. HER2-Positive BC

On the basis of the aforementioned preclinical data, the clinical activity of CDK4/6 inhibitors in HER2-positive BC was explored. The combination of palbociclib, fulvestrant and trastuzumab and pertuzumab was investigated in the neoadjuvant setting in the phase II, single arm, NA-PHER2 trial, that showed a significant effect on the expression of ki67 after 2 weeks of treatment and at time of surgery; this result suggests that triple targeting of ER, RB1, and HER2 could be an effective chemotherapy-free strategy in patients with HR-positive, HER2-positive BC [111]. In the metastatic setting, several trials are exploring the synergistic antitumor activity of dual/triple blockade: the phase II PATRICIA study (NCT02947685) is testing the efficacy and the safety of palbociclib plus trastuzumab ± letrozole for the treatment of postmenopausal women who had received 2-4 prior lines of anti-HER2-based regimens [112]; the PATINA study (NCT02947685) is a phase III, open-label trial aiming at determining the effect of adding palbociclib to anti-HER2 and ET maintenance after induction of 6–8 cycles of chemotherapy (taxane or vinorelbine) and anti-HER2-therapy in the first line setting [113]. Palbociclib is also being investigated for the treatment of brain metastasis in combination with trastuzumab (NCT0274681).

In a phase I/II trial (NCT02657343) the combination of continuous low dose of ribociclib (400 mg) with anti-HER2 therapy has proven to be safe in heavily pre-treated patients, however the clinical activity of dual combination is still under investigation [114]. Ribociclib has also been investigated in combination with TDM-1 in a phase 1b study (NCT02657343), showing that the co-targeting of HER2 and CDK4/6 was well tolerated at the recommended phase 2 dose of 400 mg of ribociclib with evidence of clinical activity [115].

Recently, results from the phase II monarchHER trial, showed that the combination of abemaciclib, fulvestrant, and trastuzumab significantly improved PFS compared to standard chemotherapy plus trastuzumab (8.3 vs. 5.7 months, HR 0.67, *p* = 0.051) with a tolerable safety profile [116].

The effectiveness of CDK4/6 inhibitors in HER2-positive BC is still under exploration, however, results from phase II studies suggest that a chemotherapy-free regimen might be a possible treatment option for these patients.

### 6.4. Combination with Immunotherapy and Other Agents

A growing number of trials are exploring the combination of CDK4/6 with other targeted therapies and with immunotherapy. The majority of novel combinations involve the PI3K pathway. The phase I/II trial, TRINITI-1 (NCT02732119) investigated the triplet therapy, exemestane plus ribociclib plus everolimus, after progression on CDK4/6 plus ET therapy; results showed an ORR and PFS lower than that seen in BOLERO-2 trial of exemestane plus everolimus (ORR 8% vs. 9.5% and median PFS of 5.7 vs. 6.9 months in BOLERO-2 trial) [117,118]. This difference could reflect the different endocrine sensitivity of patients enrolled as well as the prevalence of PIK3CA and SR1 mutations which were present in 30% of patients enrolled in TRINITI-1 trial [118]. Two phase II trials, MAINTAIN (NCT02632045) and PACE (NCT03147287), are evaluating the efficacy of CDK4/6i (ribociclib and palbociclib, respectively) plus fulvestrant in HR-positive BC patients who progressed after a CK4/6i regimen; the PACE study is also exploring the synergistic effect of addition of avelumab (anti-PD-L1) [119,120].

Ongoing trials are also evaluating the role of CDK4/6 inhibition in TNBC; in this setting RB1 loss is quite frequent, making the use of CDK4/6i more challenging and highlighting the importance of selecting specific subset of patients that might be sensitive to CDK4/6 inhibition. Particularly, patients with androgen receptor (AR)-positive TNBC seem to be more sensitive to this combination strategy, due to the association between the expression of AR and RB1 [121]. Actually, two phase I/II trials are evaluating the combination of bicalutamide (AR inhibitor) and palbociclib or ribociclib in AR+ TNBC to test the hypothesis that androgen blockade, paired with CDK4/6 inhibition would improve the efficacy in androgen-dependent BC (NCT02605486, NCT03090165); preliminary results showed that the combination strategy was well tolerated with no unexpected toxicity observed [89,122,123].

## 7. Mechanisms of Resistance and Potential Biomarkers of Response

Several studies are exploring the mechanisms of resistance to CDK4/6i, focusing on two main categories of alterations which involve the cell cycle machinery and the upstream signaling, such as MAPK pathway and PI3K/Akt/mTOR pathway.

RB is the primary phosphorylation target of CDK4/6 and the presence of phosphorylated RB (pRB) is an important biomarker. Preclinical studies showed that loss of *RB1* is associated with de-novo resistance to CDK4/6i and loss of *RB1* over time, during prolonged exposure to palbociclib or ribociclib, has been described and associated with acquired resistance. In addition, the presence of acquired mutations in RB1, identified in circulating tumor DNA (ctDNA), has also been associated to clinical resistance in BC patients treated with CDK4/6i [124,125,126,127]. The complex cyclin E/CDK2 may also be useful as biomarker, as described in an analysis of PALOMA-2 and PALOMA-3 studies, showing that high expression of cyclin E1 (*CCNE1*) mRNA was associated with shorter PFS in pre-treated patients (cohort from PALOMA-3) but not in previously untreated patients (cohort from PALOMA-2) [128,129]. Acquired CDK6 amplification promotes CDK4/6i resistance through reduced expression of estrogen receptor (ER) [130]. The role of p16 amplification as biomarker remains controversial since results from biomarker analysis of PALOMA-1, PALOMA-2, and PALOMA-3 did not show any significant difference in terms of PFS in p16/CCND1 cohort compared with unselected cohort [128,129,131].

The upstream signal transduction alterations can also be involved in promoting resistance to CDK4/6i; alterations in *AKT1, KRAS, HRAS. NRAS, FGFR2*, and *ERBB2* have been associated to CDK4/6i resistance and/or antiestrogen resistance in whole exome analysis of tumor specimens [132]. Also point mutations in ERBB2 have been associated with palbociclib and antiestrogen resistance; this resistance can, in vitro, be reverted by adding the HER2 kinase inhibitor neratinib [133,134]. The prospective, pharmacogenetic study ECLIPS is identifying predictive biomarkers in response to palbociclib plus ET combination treatment; results showed that the thymidine kinase-1 (TK1), key regulator of S/G2 phase, was significantly increased before treatment when compared after 3 months of treatment in patients who experienced disease progression, suggesting that TIK1 mRNA copies/mL can be correlated to acquired resistance to CDK4/6i [135]. Gene analysis results from 348 HR-positive HER2-negative metastatic BC samples showed that loss of FAT1, a tumor suppressor belonging to chaderin family involved in upregulation of CDK6 expression via Hippo pathway, is associated with a shorter PFS, suggesting its possible role as predictor of CDK4/6i resistance [136].

Finally, the ongoing phase IIIb trial, Bioitalee (NCT03439046), is studying ctDNA alterations and their evolution during treatment with ribociclib and letrozole as first line treatment: of 287 post-menopausal patients enrolled in the study, samples from 271 patients were considered suitable for a preliminary biomarker analysis, the main findings showed that the most frequently altered genes were *PIK3CA* (22.14%), *TP53* (15.50%), *FGFR1* (6.64%), *CCND1* (4.80%), *CCND2* (4.80%), *KMT2C* (4.43%), *MYC* (4.06%), *CDK4* (3.69%) *AKT1* (3.32%), *PTEN* (3.32%), *ERBB2* (2.58%), *CCND3* (2.58%), *APC* (2.21%) and *MAP2K4* (2.21%). More than one alteration was observed in 28% pts (either CNV or hotspots). Mutation in *KTM2C* or alterations in genes belonging to the “estrogen receptor nuclear function” (ERnf) pathway (i.e., *KTM2C*, *ESR1*, *GATA3*, and *MYC*) are more frequent in patients with recurrent disease versus de novo advanced BC, while copy number gain of FGFRs (FGFR1, 2 and 3) are prevalent in patients with more aggressive disease. Patients with early disease progression had more frequently alterations in genes of the HER family and CDK4/6 pathway, TP53 mutations and *MYC* gain, suggesting these biomarkers as potential markers of intrinsic resistance to first-line treatment with ribociclib and letrozole. Final biomarker dynamics and pharmacogenomics analysis are still ongoing [137].

## 8. Conclusions

The introduction of CDK4/6 inhibitors in clinical practice represents a major advancement for the treatment of HR-positive HER2-negative BC (Table 1). The combination of CDK4/6i and ET seems to be effective in all clinicopathological subgroups, as emerged by the large pooled analysis of FDA [138]. Preclinical and translational research is now exploring the heterogeneous landscape able to drive the response to these agents. In addition, clinical studies are investigating novel combination strategies in other BC subtypes, such as HER2-positive BC and TBNC, and are focusing on post-progression strategies after CDK4/6 inhibition. Based on these preclinical and clinical data, there is a strong evidence showing that CDK4/6 inhibition can provide therapeutic benefit, enhancing the antitumor activity of other class of agents, such standard chemotherapy regimen, immunotherapy regimens, targeted therapies (Table 1) [139].

## Figures and Tables

**Table 1:** Summary of randomized, phase II/III, clinical trials evaluating CDK4/6 inhibitors in BC.

Trial Name	Phase	Setting	Population	Treatment Arms	Sample Size	Primary Outcome (Exp vs. Ctrl Arm) HR (95% CI)
**MONALEESA-2**	III	Advanced or Metastatic	AI-sensitive postmenopausal women with HR-positive/HER2-negative advanced or metastatic BC; no previous systemic therapy for ABC	Ribociclib + Letrozole vs. Letrozole + Placebo	668	PFS 25.3 vs. 16 months (HR 0.568; 95% CI 0.457–0.704)
**MONALEESA-3**	III	Advanced or Metastatic	AI-sensitive/resistant postmenopausal women with HR-positive/HER2-negative advanced or metastatic BC; 0-1 line of ET for ABC	Ribociclib + Fulvestrant vs. Fulvestrant + Placebo	726	PFS 20.5 vs. 12.8 months (HR 0.593; 95% CI 0.480–0.732)
**MONALEESA-7**	III	Advanced or Metastatic	AI-sensitive peri/premenopausal women with HR-positive/HER2-negative advanced or metastatic BC; no previous ET and up to 1 line of CT for ABC	Ribociclib + TAM/NSAI vs. TAM or NSAI + Placebo	672	PFS 23.8 vs. 13.3 months (HR 0.553: 95% CI 0.441–0.694)
**MONARCH-2**	III	Advanced or Metastatic	AI-resistant pre/postmenopausal women with HR-positive/HER2-negative advanced BC that progressed after ET; no previous CT for ABC	Abemaciclib + Fulvestrant vs. Placebo + Fulvestrant	669	PFS 16.4 vs. 9.3 months (HR 0.553; 95% CI 0.449–0.681)
**MONARCH-3**	III	Advanced or Metastatic	AI-sensitive postmenopausal women with HR-positive/HER2-negative advanced or metastatic BC; no previous systemic therapy for ABC	Abemaciclib + NSAI vs. Placebo + NSAI	493	PFS 28.1 vs. 14.7 months (HR 0.540; CI 0.418–0.698)
**MonarchHER**	III	Advanced or Metastatic	Postmenopausal women with HR-positive/HER2-positive locally advanced or metastatic BC who have previously received at least 2 HER2-directed therapies for advanced disease	A. Abemaciclib + Trastuzumab + Fulvestrant B. Abemaciclib + Trastuzumab C. Trastuzumab + SOC CT	237	PFS 8.3 vs. 5.7 (A vs. C) (HR 0.673; *p* = 0.05)
**PALOMA-1/TRIO-18**	II	Advanced or Metastatic	AI-sensitive postmenopausal women with HR-positive/HER2-negative advanced or metastatic BC; no previous systemic therapy for ABC	Palbociclib + Letrozole vs. Letrozole	165	PFS 20.2 vs. 10.2 months (HR 0.488; 95% CI 0.319–0.748)
**PALOMA-2**	III	Advanced or Metastatic	AI-sensitive postmenopausal women with HR-positive/HER2-negative advanced or metastatic BC; no previous systemic therapy for ABC	Palbociclib + Letrozole vs. Letrozole	666	PFS 27.6 vs. 14.5 months (HR 0.563; 95% CI 0.461–0.687)
**PALOMA-3**	III	Advanced or Metastatic	AI-resistant pre/postmenopausal women with HR-positive/HER2-negative advanced or metastatic breast cancer that progressed after ET	Palbociclib + Fulvestrant vs. Fulvestrant + Placebo	521	PFS 9.5 vs. 4.6 months (HR 0.46; 95% CI 0.36–0.59)
**PEARL**	III	Advanced or Metastatic	AI-resistant postmenopausal women with HR-positive, HER2-negative metastatic BC	Palbociclib + ET vs. Capecitabine	601	PFS 7.5 vs. 10 months (HR 1.09; 95% CI 0.83, 1.44)
**FELINE**	II	Neoadjuvant	Postmenopausal women with HR-positive/HER2-negative early BC	Ribociclib 600 + Letrozole vs. Ribociclib 400 + Letrozole vs. Ribociclib + Placebo	121	PEPI score 0 at surgery 25.8% vs. 25.4% (*p* = 0.96)
**neoMONARCH**	II	Neoadjuvant	Postmenopausal women with stage I/IIIB HR-positive/HER2-negative early BC	A. Abemaciclib + Anastrozole B. Abemaciclib alone C. Anastrozole	224	Ki67 change 58–68% (A-B) vs. 14% (C) (*p* < 0.001)
**PALLET**	II	Neoadjuvant	Postmenopausal women with HR-positive/HER2-negative early BC	A. L B. L then L + P C. P then L + P D. L + P	306	CRR 54.3% vs. 49.5% (*p* = 0.20)
**CORALEEN**	II	Neoadjuvant	Postmenopausal women with stage I-IIIA HR-positive, HER2-negative, luminal B early BC	Ribociclib + Letrozole vs. Chemotherapy	106	PAM 50 low ROR at surgery 46.9% vs. 46.1% (95% CI 32.5–61.7 vs. 32.9–61.5)
**NeoPal**	III	Neoadjuvant	Postmenopausal women with II-IIIA PAM 50 ROR-defined Low or Intermediate Risk, HR-positive, HER2-negative early BC	Palbociclib + Letrozole vs. Chemotherapy	106	RBC 0–1 7.7% vs. 15.7% (95% CI 0.4–14.9 vs. 5.7–25.7)
**MONARCH-E**	III	Adjuvant	Pre/postmenopausal women or men with high-risk node-positive HR-positive, HER2-negative early BC	Abemaciclib + ET vs. ET	5637	iDFS (results awaited)
**PENELOPE-B**	III	Adjuvant	Pre/postmenopausal women with HR-positive/HER2-negative early BC at high risk of relapse after showing no pathological complete response to neoadjuvant chemotherapy	Palbociclib vs. Placebo	1250	iDFS (results awaited)
**PALLAS**	III	Adjuvant	Pre/postmenopausal women or men with stage II/III HR-positive/HER2-negative early BC at moderate to high risk of recurrence	Palbociclib (2 y) + ET (5 y) vs. ET for 5 years	5796	iDFS (results awaited)
**MAINTAIN**	II	Advanced or Metastatic	Pre/postmenopausal women or men with HR-positive/HER2-negative advanced or metastatic BC who have progressed on an AI plus a CDK4/6 inhibitor (either palbociclib or ribociclib)	Ribociclib + Fulvestrant vs. Fulvestrant + Placebo	132 (estimated)	24-wk PFS (results awaited)
**PACE**	II	Advanced or Metastatic	Pre/postmenopausal women or men with HR-positive/HER2-negative advanced or metastatic BC who have progressed on an ET plus a CDK4/6 inhibitor and up to 1 line of CT for ABC	Palbociclib + Fulvestrant vs. Palbociclib + Fulvestrant + Avelumab vs. Fulvestrant	220 (estimated)	PFS (results awaited)
**PATINA**	III	Advanced or Metastatic	Patients with HR-positive, HER2-positive metastatic BC who received induction treatment as first line therapy	Palbociclib + Anti-HER2 + ET vs. Anti-HER2 + ET	496 (estimated)	PFS (results awaited)
**NATALEE**	III	Adjuvant	Pre/postmenopausal women or men with HR-positive/HER2-negative early BC	Ribociclib + ET vs. ET	4000 (estimated)	iDFS (results awaited)

Abbreviations: Exp—experimental; Ctrl—control; HR—hazard ratio; CI—confidence interval; AI—aromatase inhibitors; HR—hormone receptor; HER2—human epidermal growth factor 2; BC—breast cancer; ABC—advanced breast cancer; PFS—progression free survival; ET—endocrine therapy; CT—chemotherapy; TAM—tamoxifen; NSAI—non steroidal aromatase inhibitors; SOC—standard of care; L—letrozole; P—palbociclib; iDFS—invasive disease free survival; ROR—risk of recurrence; CRR—clinical response rate; RCB—residual cancer burden.

## Abbreviations

AE	Adverse event
AIs	Aromatase Inhibitors
AR	Androgen receptor
BC	Breast Cancer
CDK 4/6	Cyclin-Dependent Kinase 4/6
CDKi	CDK inhibitor
CIs	Confidence Intervals
CIP	CDK-interacting protein
EMA	European Medicine Agency
ER	Estrogen Receptor
ET	Endocrine Therapy
FDA	Food and Drug Administration
HER2	Human Epidermal Growth Factor Receptor 2
HR	Hormone Receptor
INK4	inhibitor of CDK4
KIP	CDK-kinase inhibitory protein
LAR	Luminal androgen receptor
MEK	Mitogen-activated protein kinase kinase
mTOR	Mammalian Target Of Rapamycin
PD-L1	Programmed Cell Death Ligand-1
PI3K	Phosphatidylinositol 3-kinase
PIK3	Phosphoinositide 3-kinase
pRB	Retinoblastoma-associated proteins
NSAI	Non-Steroidal Aromatase Inhibitors
ORR	Objective Response Rate
OS	Overall Survival
PFS	Progression Free Survival
RBC	Residual Cancer Burden
TNBC	Triple negative breast cancer
Treg	Regulatory T cells
